# Recovery of Metals from Heat-Treated Printed Circuit Boards via an Enhanced Gravity Concentrator and High-Gradient Magnetic Separator

**DOI:** 10.3390/ma14164566

**Published:** 2021-08-14

**Authors:** Yushuai Xian, Youjun Tao, Fangyuan Ma, You Zhou

**Affiliations:** 1 Key Laboratory of Coal Processing and Efficient Utilization of Ministry of Education, China University of Mining and Technology, Xuzhou 221116, China; tb18040033b4@cumt.edu.cn (Y.X.); zhouyou2010@csu.edu.cn (Y.Z.); 2 School of Chemical Engineering and Technology, China University of Mining and Technology, Xuzhou 221116, China; 3 School of Metallurgy and Environment, Central South University, Changsha 410000, China

**Keywords:** printed circuit board, recovery, thermogravimetric analysis, enhanced gravity separation, high-gradient magnetic separation

## Abstract

The recovery and reuse of waste printed circuit boards (PCBs) has attracted more and more attention from global researchers, as recycling of waste PCB metals is of great significance to the rational utilization of metal material resources. This study puts forward a clean and economical method in which enhanced gravity separation and wet high-gradient magnetic separation were combined to recover waste PCBs with heat treatment at a temperature of 240 °C. The heat treatment could improve the metal liberation effect of the PCBs, and the thermal behavior was measured by thermogravimetric analysis (TGA). The pyrolysis of the non-metal fraction (NMF) began around 300 °C, and the glass transition temperature of epoxy resin was 135.17 °C. The enhanced gravity separation technique was used for the separation of metals and NMF under the compound force field. The metals grade of the gravity concentrates fraction (GRF) was 82.97% under the optimal conditions, and the metals recovery reached 90.55%. A wet high-gradient magnetic separator was applied to classify the GRF into magnetic (MA) and non-magnetic (NMA) fractions, which could achieve iron and copper enrichment. After the three stages combined process, the copper and iron grades of the NMA and MA fractions were 70.17% and 73.42%, and the recovery reached 74.02% and 78.11%, respectively.

## 1. Introduction

With the rapid development of the global electronic information industry, the requirement for electrical and electronic equipment is sharply increasing. At the same time, waste electrical and electronic equipment (WEEE) has become a kind of solid waste that cannot be ignored, and e-waste is one of the fastest growing waste streams globally [1,2,3]. About 2 million tons of waste printed circuit boards (PCBs), as part of e-waste with excellent recovery value, are discarded annually worldwide [4], assuming that PCBs are 5% of e-waste [5]. The bare PCB is composed of metals, woven glass fiber and flame retardant [6,7], especially, as some metals are more abundant than in raw material sources [8,9], PCB recovery is called “urban mining” [10,11]. This is exemplified by studies showing that the copper content in PCBs is about 20%, much higher than the 0.2–0.7% found in copper ore [12,13,14]. 

The main mechanical-physical methods for PCB recovery include magnetic separation [15,16], gravity separation [17,18], electrostatic separation [19,20], and vacuum metallurgy [21,22]. The high-gradient magnetic separator has been applied to the separation of paramagnetic iron elements from metal concentrates [23,24], which could obtain the magnetic (MA) and non-magnetic (NMA) metal products from waste PCBs. Gravity separation could be used for metals enrichment due to the density differences between the non-metallic fraction (NMF) and metals. In addition, the enhanced gravity separation was utilized to recover the metals from PCBs. Duan et al. and Ma et al. have used the Falcon enhanced gravity concentrator to classify the metals of PCBs and achieved excellent results [17,18]. Electrostatic separation technique utilizes the different electrical properties of the NMF and metals, whereby the organic particles were negatively charged while the metal particles are positively charged during the triboelectrification process. These mechanical-physical methods have the advantage of being economical and generating less secondary pollution. However, the recovered metals are generally compounded and need further treatment for single metal recovery. 

In this paper, the mechanical-physical approaches of enhanced gravity separation and high-gradient magnetic separation were combined to recover the metals from waste computer PCBs. The heat treatment of waste PCBs was performed before the recovery tests. The glass transition temperature of epoxy resin of PCB was measured by the thermogravimetric analysis (TGA) method [25], and analyzed by DTA and TMA curves [26,27]. The morphology of PCB was observed by SEM after heat treatment. Based on the premise of an improved liberation effect from PCBs after heat treatment, the enhanced gravity concentrator and high-gradient magnetic separator were used for metals separation in succession. This is the first study of a combined process including heat treatment, enhanced gravity separation and wet high-gradient magnetic separation for metal recovery and the enrichment of copper and iron. It provides an economical, efficient and environmentally friendly method for the recycling of waste PCBs and provides significant guidance for the recycling of metal materials. 

## 2. Materials and Methods

### 2.1. Materials

Fifty kg of waste computer motherboards were purchased from a solid waste recovery station in Xuzhou (Jiangsu, China). The PCBs are FR-4 multilayered structures boards. These waste PCBs were disassembled manually to remove the electronic components, and the representative sample was collected by the cone and quarter method after liberation by a hammer crusher (KERP-180X150B, Huayu, Zhengzhou, China). Before the PCB heat treatment and separation experiments, the elemental composition should be confirmed. For this a sample was ground to less than 0.074 mm by using a ring pulverizer (FW400A, Maike, Taian, China), and dried in the oven (105 °C) for two hours, then the elemental composition of the waste PCBs was analyzed by XRF (S8 Tiger, Bruker, Karlsruhe, Germany) with the elements analysis ranging from ^4^Be to ^92^U. 

### 2.2. Methods

#### 2.2.1. Heat Treatment 

Epoxy resin is the adhesive of PCB, and its glass transition and thermal decomposition occur at a high temperature [26,27].The waste PCBs was subjected to heat treatment for the better metal liberation, but thermal decomposition should be avoided due to the potential release of noxious gases [28,29,30]. Keeping the heat temperature between the glass transition and thermal decomposition ones is the ideal condition to achieve an improved liberation. 

The PCB samples were ground to under 0.074 mm, and 15–20 mg were weighed for the thermogravimetric analysis (DSC-TGA, STA 449F5, Netzsch, Bavaria, Germany) [26,27]. The heating rate was 10 °C/min, and heating temperature ranged from 25 °C to 1000 °C with a nitrogen atmosphere. Meanwhile, the glass transition temperature of epoxy resin could be measured by the TMA curve [26], which was produced by heating from 25 to 200 °C at a rate of 5 °C/min in a crucible with 15–20 mg of sample under a nitrogen atmosphere. The heat treatment test was performed in a conventional muffle furnace, and pieces of PCB (20 × 20 mm) were placed into the muffle furnace with a fixed temperature. The heated PCB was broken by a hammer crusher and the liberation products were observed by polarizing microscope (BA310Pol, Maikeaodi, Xiamen, China). SEM (Quanta 250, FEI, Hillsboro, OK, USA) was employed to observe the progressive change of PCB before and after heat treatment. The electron gun of the SEM instrument is a tungsten filament with a current probe of 1 pA–2 μA, and the magnification is from 2 to 1 million times. 

#### 2.2.2. Enhanced Gravity Separation

The enhanced gravity concentrator (Falcon SB40, FALCON, Langley, British Columbia, Canada) can stratify particles with different densities under its powerful centrifugal force in a flowing film [18]. After the heat treatment, the PCB was crushed to −0.5 mm and separated by the Falcon concentrator. The feed concentration was set at 100 g/L and the PCB particles were drawn into the Falcon concentrator by a peristaltic pump after mixing evenly. The adjustable parameters of the device include the rotational speed of the drum (*ω*), the jet pressure (*P*) and the feed speed (*Q*). The Box-Behnken response surface methodology was implemented to analyze the interaction of the three parameters and proposed the optimization experimental conditions for metals recovery from waste PCBs. The gravity concentrates fraction (GRF) was collected and analyzed by XRF and XRD after the enhanced gravity separation. It is worth noting that each element is calibrated before the XRF test process to ensure the high accuracy of the metal content in the sample. In addition, precious metals such as gold, silver are tested by atomic absorption spectrometry (AAnalyst200, PerkinElmer, Waltham, NA, USA).

#### 2.2.3. High-Gradient Magnetic Separation 

Considering the high iron content in the PCB samples, the iron element with its paramagnetic property could be separated through the utilization of a high-gradient magnetic separation technique [23,24]. A wet high-gradient magnetic separator (Slon-100, Jinhuan, Ganzhou, China) was adopted to separate the GRF by enhanced gravity separation. The magnetic field intensity of high-gradient magnetic separator reached 1.228 T, which could classify the weak magnetic metals in the PCBs. The GRF was drawn into the high-gradient magnetic separator after the adjustment of the pulsion stroke, the MA and NMA fractions were collected and an XRF test was employed to measure the elemental composition of the MA and NMA fractions. The experimental parameters of the high-gradient magnetic separator include the magnetic flux density (*B*), pulse intensity (*i*) and particle size (*d*), which were explored in an orthogonal test with three levels.

### 2.3. X-ray Diffraction Tests

In order to clarify the effect of each process on the recovery of metals, XRD (D8 ADVANCE, Bruker, Karlsruhe, Germany) tests were carried out to determine the phase composition of the raw sample, GRF, MA and NMA fractions. The PCB samples and the products at each stage were collected and ground to below 0.074 mm, dried for 2 h at 105 °C, and the phase composition was analyzed by their XRD patterns. The heat treatment was focused on the glass transition of the epoxy resin, which occurred before the thermal decomposition process. Therefore, it is considered that the phase composition of the PCB was unchanged after heat treatment.

## 3. Results and Discussion

### 3.1. Properties of Materials

The elemental composition of the PCB samples is presented in Table 1, where it can be seen that the composition coincided with that of computer PCBs given in the literature [23,31,32], which mainly composed of copper, oxygen, silicon and iron, etc. Nonmetallic elements such as oxygen and silicon mainly existed in the NMF, the present of bromine was due to the flame retardant of the epoxy resin. The metal elements copper, iron, aluminum and lead were mainly present as elementary substances, which come from the copper foils, wire and electronic components, and the zinc and nickel were expressed as alloys in the PCBs [33]. The calcium, magnesium and part of aluminum are attributed to the mineral admixture in the PCB. The content of copper in the PCB sample reached 28.55%, much higher than that of the raw ore, which coincides with the concept of “urban mining”. The recovery of PCBs is the key link of valuable metals from waste to reuse.

### 3.2. Heat Treatment

#### 3.2.1. Weight Loss Characteristics of PCBs

**Figure 1:**
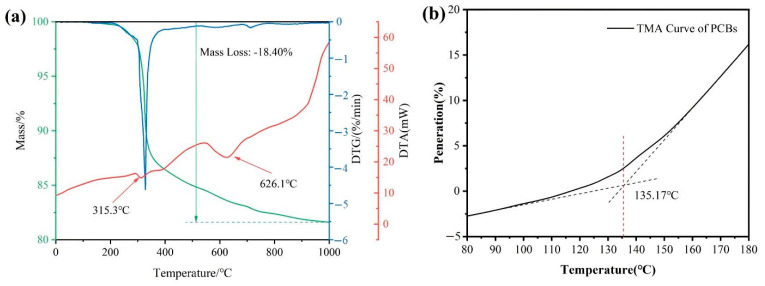
Characteristics of PCB after heat treatment. (**a**) TG-DTA curves of brominated epoxy resins in the PCBs. (**b**) TMA curve of brominated epoxy resins.

Epoxy resin will be damaged during the thermal decomposition process, which will release noxious gases, such as polychlorinated dibenzo-*p*-dioxins (PCDD) and polychlorinated dibenzofurans (PCDF) [34,35]. The pyrolysis behavior of the PCB sample was studied by thermogravimetric analysis and the results presented in Figure 1. 

According to the DTA curve, the weight loss of NMF occurred at two stages, the first peak appearing at 315.3 °C and beginning around 300 °C, and the second peak emerging at 626.1 °C, indicating that the thermal decomposition of organic matter occurred near these temperatures. According to the studies in the literature [27] the rapid thermal decomposition of epoxy resin occurs above 300 °C, followed by slow combustion after 500 °C. The weight loss curve shows that the weight reduction of NMF was 18.40% in the thermogravimetric process. As the thermal decomposition starts at 300 °C, the temperature of the heat treatment should be controlled below this temperature to avoid the decomposition of the epoxy resin. The TMA curve revealed that the glass transition temperature of the brominated epoxy resin was 135.17 °C in PCB samples. If the heating temperature of the PCBs was lower than the glass transition temperature, the brominated epoxy resin is frozen [26]. The heating temperature should be higher than 135.17 °C to unfreeze the epoxy resin chains and realize the structural relaxation of PCBs. 

#### 3.2.2. Degree of Liberation

**Figure 2:**
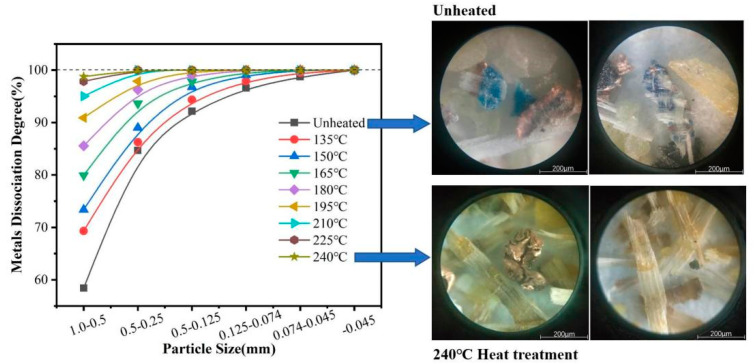
Comparison of the metals liberation degree of PCBs with different heating temperatures.

A comparison of the degree of metals liberation under different heating temperatures is shown in Figure 2, where three points can be seen: (1) When the heating temperature is higher than 135 °C, the degree of metals liberation of the PCBs increased significantly compared with the unheated sample, and the improvement of metals liberation of PCBs is obvious after the heat treatment. (2) As the temperature rises gradually, the metal liberation effect of each particle size increases in an orderly way (especially for the +0.5 mm particle size). (3) When the temperature is above 240 °C, the metals liberation effect of PCBs is basically saturated and the thermal decomposition temperature was not reached at this time. From Figure 2, the liberation products of PCBs are clearly divided to metals and NMF with a heating temperature of 240 °C, and the layers of fiber glass are all stripped well from each other due to the glass transition temperature of epoxy resin. 

#### 3.2.3. Micro Characteristics

**Figure 3:**
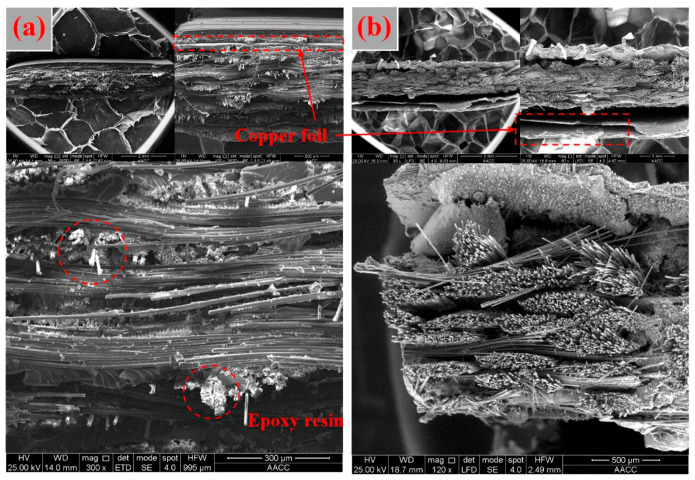
The SEM of the morphology of unheated (**a**) and heat treated (**b**) PCBs.

Figure 3 shows the morphology of PCB after the heat treatment at 240 °C. It can be seen from Figure 3a that the support structure of PCBs was composed of fiberglass with an epoxy resin adhesive and copper foils covered both sides of the fiberglass [34]. Comparing Figure 3a,b, it can be seen that the white epoxy resin is almost invisible after heat treatment, and fiberglass of the PCBs was loose and scattered, indicating that the glass transition of epoxy resin has been occurred during the heating process [26]. In addition, an obvious gap appeared between copper foils and fiber glass, resulting in the metals of PCBs being more easily released during in the liberation process. 

#### 3.2.4. Mechanical Characteristics

**Figure 4:**
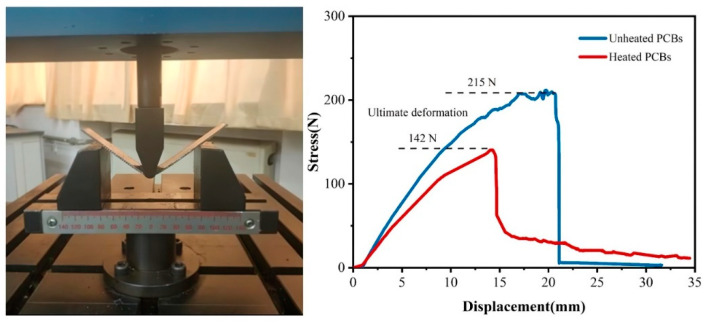
Measurement results of mechanical characteristics.

The mechanical characteristics of heated PCBs are shown in Figure 4. It can be seen that the ultimate deformation of heated PCB (at 240 °C) is lower than that of unheated board under the gradually increasing external stress. The deformation displacement of the heated PCB was 14 mm, however, the deformation displacement of an untreated board was increased to 21 mm. Besides, the heated board was fractured when the compressive stress reached 142 N, which is lower than the unheated board at 215 N. Combining the SEM tests, it can be observed that the unfrozen epoxy resin cannot maintain the fiberglass and copper foils stuck tightly together after the glass transition. The heat treatment of PCBs not only reduces the energy consumption during the crushing process, but also improves the metals liberation effect. 

### 3.3. Enhanced Gravity Separation Results 

#### 3.3.1. Orthogonal Enhanced Gravity Separation Experiments 

The experimental design of the orthogonal test was shown in Table 2. The Box-Behnken response surface methodology was utilized to analyse the interaction between the three parameters. The quadratic model was recommended to fit the orthogonal results, which the value of Prob > F lower than 0.0001 [36]. In this case A, B, BC and B^2^ are significant terms in the metals recovery with Prob > F values under 0.05. The final equation in terms of coded factors was as follows:

(1) $$\begin{array}{cc} \mathrm{Metals}\;\mathrm{Grade}\;=\; & 90.03-0.05\times A-347.08\times B+0.15\times C-0.68\times A\times B\\ - & 0.000255\times A\times C+1.18\times B\times C+0.000185\times A^{2}+9197.5\times B^{2}\\ - & 0.000425\times C^{2} \end{array}$$

The goodness of the recommended quadratic model is analyzed in Table 3, where the value of the prediction R^2^ was 0.8988, which means that the second-order polynomial model was accurate. The predicted residual sum of squares measures the signal to noise ratio, which is desirable to be greater than 4 [35,36]. The value of 22.825 indicates an adequate signal, which can be used to navigate the design space.

**Figure 5:**
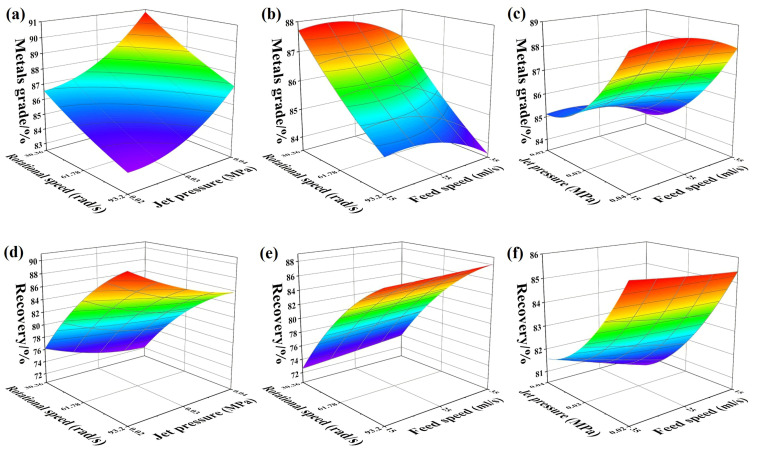
The response surfaces of the experimental factors of centrifugal separation. The relationship between metals grade and test conditions was shown in (**a**–**c**); and the relationship between recovery and test conditions was shown in (**d**–**f**).

The response surface of the three parameters: rotational speed (*ω*), jet pressure (*P*) and feed speed (*Q*) is shown in Figure 5. It can be seen in Figure 5a that the increase of rotational speed could promote the increase of metals recovery. Centrifugal intensity contributes to the settling force of particles in the flow film, so an increase of the settling force makes the metals particles more stable at the bottom of the flow film and not hindered by the anti-settling force in the compound force field. In Figure 5b, it can be seen that jet pressure of the water flow has a negative effect on the increase of metals recovery, but the jet flow can prevent the deposition of NMF with low density, which is very important for the improvement of the metals grade. Meanwhile, the increase of feed speed caused the enlargement of the volume fraction of PCB particles fed per unit time, which leads to the a weakening of the separation accuracy and worse separation conditions in the flow film. The orthogonal test of enhanced gravity separation was optimized by response surface methodology (see Table 4), and the metals grade and recovery of gravity concentrates reached 82.97% and 90.55%, respectively. The metal element distribution of the concentrates and tailings of the enhanced gravity separation is shown in Table 5, where it can be seen that the different density metals were also separated, as the the low density metals (Al, Mg, Ca) were found in the tailings, and the heavy density metals (Cu, Fe, Sn, Zn, Ag et al.) were present in the enhanced gravity separation concentrates.

#### 3.3.2. XRD Analysis

**Figure 6:**
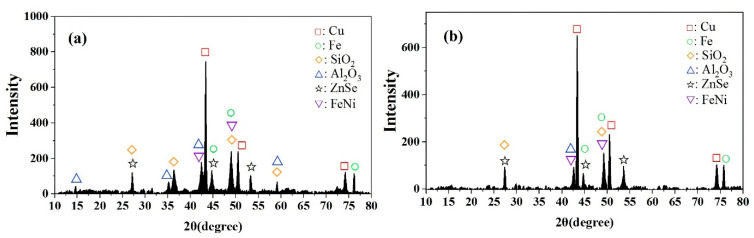
XRD patterns of PCB sample (**a**) and GRF (**b**).

The XRD patterns of raw PCBs and enhanced gravity concentrates (GRF) are shown in Figure 6, where it can be seen that the most obvious peak of the PCB sample corresponds to elemental copper, which is consistent with the high copper content in the XRF results. The peak amplitude of elemental iron is lower than that of copper, which is mainly due to the presence of iron in elementary substance form and FeNi alloy. Zinc and aluminum also appeared in the XRD patterns of the PCB samples. ZnSe was present as an admixture of semiconductors. Aluminum was present as aluminum oxide, and silicon dioxide was obviously present in the XRD patterns. These substances are deposited as mineral admixtures in the ceramics, electronic components and glass fibers [33]. From Figure 6b, the mineral peaks were changed after enhanced gravity separation. The metals elements peaks were still observed, but the aluminum oxide and silicon dioxide peaks disappeared in the GRF, indicating that the metals and alloys were enriched by the compound force field of the Falcon concentrator.

### 3.4. High-Gradient Magnetic Separation 

#### 3.4.1. Orthogonal High-Gradient Magnetic Separation Experiments

**Figure 7:**
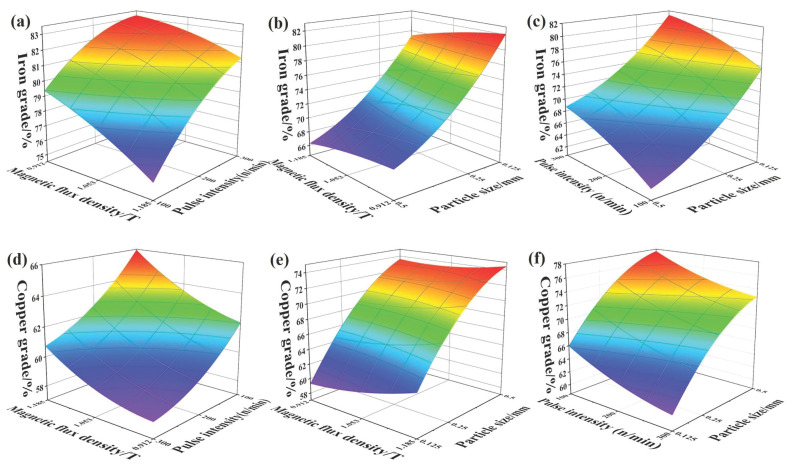
The response surfaces of the experimental factors of high-gradient magnetic separation. The relationship between iron grade and test conditions was shown in (**a**–**c**); and the relationship between copper grade and test conditions was shown in (**d**–**f**).

From the XRD results (Figure 6), the element iron was present as iron elementary substance and FeNi alloy with paramagnetic and weak magnetic properties. The high-gradient magnetic separation can not only purify elemental iron, but also enhance the copper content in the metal concentrates. The orthogonal test of high-gradient magnetic separation was implemented by response surface methodology, as shown in Figure 7. The three experimental design parameters were magnetic flux density (*B*), pulse intensity (*i*) and particle size (*d*) and are listed in Table 6. The orthogonal result was also analyzed by a quadratic model. The significant model terms were A, B, C and B^2^ with values of Prob > F lower than 0.05. The fitting quadratic model of iron and copper grade was as follows:

(2) $$\mathrm{Iron}\;\mathrm{Grade}=79.61-1.37\times A+2.69\times B-4.47\times C+0.46\times A\times B+0.083\times A\times C+0.097\times B\times C-0.34\times A^{2}-0.83\times B^{2}+0.39\times C^{2}$$

(3) $$\mathrm{Copper}\;\mathrm{Grade}=68.92-1.35\times A-3.04\times B+4.67\times C-0.41\times A\times B-0.28\times A\times C+0.046\times B\times C+0.57\times A^{2}+0.73\times B^{2}-0.47\times C^{2}$$

The goodness of fit of the recommended quadratic model is analyzed in Table 7. The difference between the values of the corrected R^2^ and predicted R^2^ was less than 0.2, which means that the quadratic model was reasonable. The value of the predicted residual sum of squares was 39.072, greater than the desirable value of 4 [35,36]. 

The response surfaces of the experimental conditions are shown in Figure 7, where it can be seen that the increase of magnetic flux density decreased the iron grade in the MA fraction, because the separation behavior was direct impacted by the intensity of the magnetic field, and a rising magnetic flux density leads to a greater magnetic force and more weak magnetic impurities are entrained in the MA fraction. Meanwhile, the decrease of particle size could promote the rise of the iron grade, so the full dissociation of GRF benefited from the release of magnetic iron particles. The pulse flow could remove the NMA particles with the washing water, and the increase of pulse intensity leads to an improvement of the iron grade of the MA fraction. The copper grade of NMA fraction was changed opposite to the change trend of iron grade, because they are enriched in the different fractions of the high-gradient magnetic separation products.

The optimal conditions of high-gradient magnetic separation tests are listed in Table 8. When the experimental factors were set as magnetic flux density of 0.912 T, pulse intensity of 100 n/min and particle size below 0.25 mm, the iron grade of MA was 73.42%, and the copper content of NMA reached 70.17%. Through the elemental distributions of the NMA and MA fractions of the high-gradient magnetic separation products (listed in Table 9), it can be seen that the grades of copper and iron were obviously improved through the combined process of enhanced gravity separation and high-gradient magnetic separation.

#### 3.4.2. XRD Analysis

**Figure 8:**
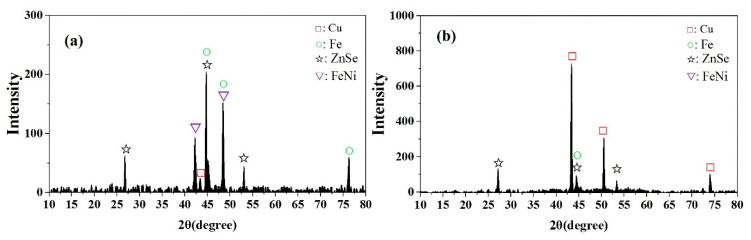
XRD patterns of high-gradient magnetic separation concentrates MA (**a**) and tailings NMA (**b**).

The XRD patterns of the high-gradient magnetic separation products are shown in Figure 8. In the MA fraction, the most obvious characteristic mineral peak belongs to the iron element, and the copper characteristic peak was significantly reduced compared with the GRF. There are also the characteristic peaks of copper and zinc in the XRD pattern of the MA fraction, indicating that the metals in the GRF are not fully dissociated and alloys with magnetic properties remained. Figure 8b shows the XRD pattern of the NMA fraction, where it is observed that the characteristic peak of elemental iron element had almost disappeared, and the dominant characteristic peak corresponded to elemental copper. 

### 3.5. Results of the Combined Process

**Figure 9:**
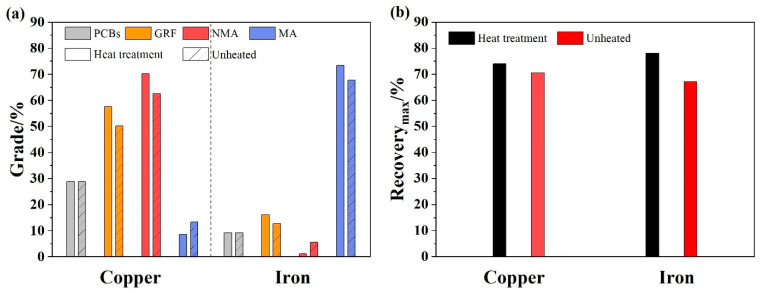
(**a**) The grade of copper and iron of each stage in the combined process with heat treatment and unheated (grey diagonal stripe) conditions; (**b**) The copper and iron recovery of the combined process.

The combined process includes three stages, (1) the first stage is the heat treatment at 240 °C, which could improve the liberation effect of PCBs. (2) the second stage is the enhanced gravity separation, where the strong centrifugal force realized the recovery of metals from raw PCBs. (3) the third stage is the wet high-gradient magnetic separation, which could divide the metal concentrates to into a copper-rich fraction and an iron-rich fraction. The grade of copper and iron in each stage was shown in Figure 9a (GRF: gravity separation concentrates fraction; MA and NMA: high-gradient magnetic separation products), the copper and iron recovery of combined process with heat treatment and unheated conditions are compared in Figure 9b. 

From the results of the combined separation process, the copper grade increased to 52.65% after the enhanced gravity separation, while the increase of iron grade was relatively lower, due to the lesser iron content in the raw PCBs. The copper and iron were enriched in the NMA and MA fractions, respectively, after the high-gradient magnetic separation. The copper grade increased to 70.17% and iron grade reached 73.42%, and the recovery of copper and iron reached 74.02% and 78.11%, respectively. When the raw PCBs were not heated in the combined process, the copper and iron grade and recovery both decreased, because the presence of epoxy resin leads to the entrainment of NMF. Compared with the raw PCBs, the copper and iron grade of NMA and MA fractions were increased by 41.62 % and 64.23%, respectively, which was a great improvement, after the combined separation process. Although the enrichment of elemental copper and iron from waste PCBs was realized in this study with economical and eco-efficiency, the recovered copper and iron cannot be directly utilized as products in a market form, and further hydrometallurgical treatment is also needed to purify the recovered metals. 

## 4. Conclusions

(1) The pyrolysis temperature of PCBs was observed by the DTA curve, and is expressed by two peaks at 315.3 °C and 626.1 °C. The glass transition temperature of the brominated epoxy resin was discovered from the TMA curve at 135.17 °C. The heating temperature of PCBs should be placed between 135 °C and 315 °C. When the temperature is above 240 °C, the metals liberation of PCBs was basically saturated and the liberation products could be clearly divided into metals and NMF according to the observations by polarized light microscopy. After the heat treatment of PCBs, it can be seen that the glass transition of epoxy resin has been occurred, and the fiberglass of the PCBs became loose and scattered. 

(2) An orthogonal test of enhanced gravity separation was designed using the response surface methodology, and the quadratic model was recommended to fit the orthogonal results with a value of Prob > F lower than 0.0001. The optimized experimental conditions of centrifugal separation are a rotational speed of 93.20 rad/s, a jet flow pressure of 0.02 MPa, a feed speed of 34 mL/s, and the metals grade and recovery achieved values of 82.97% and 90.55% in the enhanced gravity concentrates fraction (GRF).

(3) The high-gradient magnetic separation is suitable for the separation of the GRF. When the factors of high-gradient magnetic separation were chosen as magnetic flux density of 0.912 T, pulse intensity of 100 n/min and particle size below 0.25 mm, the copper and iron grade was increased significantly. Compared with the raw PCBs, the grade of copper and iron improved 41.62% and 64.23% through the combined process of the heat treatment, enhanced gravity separation and high-gradient magnetic separation with the characteristics of low energy consumption, economical process and eco-friendliness. 

## Figures and Tables

**Table 1:** The element composition of PCB sample.

Element	Content (wt%)	Element	Content (wt%)	Element	Content (wt%)
Copper	28.55	Tin	6.52	Lead	0.25
Oxygen	16.70	Aluminum	5.08	Sulfur	0.25
Silicon	14.20	Zinc	2.15	Titanium	0.24
Iron	9.19	Barium	0.82	Antimony	0.13
Bromine	7.45	Nickel	0.40	Manganese	0.11
Calcium	7.19	Magnesium	0.35	Silver	0.08

Notice: No gold element was found in the sample. The content of silver is expressed as “g/t”. Because the content of silver is very low, the content of silver in the sample was tested by atomic absorption spectrometry.

**Table 2:** Test factors and levels of centrifugal separation tests.

Code	Factors	Units	Minimum	Maximum	Levels
A	Rotational speed	rad/s	30.36	93.20	3
B	Jet pressure	MPa	0.02	0.04	3
C	Feeding speed	ml/s	15	35	3

**Table 3:** Integrated evaluation of goodness of the recommended model in centrifugal separation tests.

Standard Deviation	0.41	R^2^	0.9793
Mean value	86.30	Correcting R^2^	0.9527
Deviation coefficient	0.48	Predicting R^2^	0.8988
Prediction error sum of squares	5.81	Predicting residual sum of squares	22.825

**Table 4:** The results of enhanced gravity separation under optimal conditions.

Rotational Speed (rad/s)	Jet Pressure (MPa)	Feed Speed (mL/s)	Yield (%)	Metals Grade (%)	Recovery (%)
93.20	0.02	34	52.85	82.97	90.55

**Table 5:** The elements distribution of concentrates and tailings of enhanced gravity separation.

Concentrates				Tailings			
Element	Grade/(wt%)	Element	Grade/(wt%)	Element	Grade/(wt%)	Element	Grade/(wt%)
Copper	52.65	Zinc	3.82	Copper	2.11	Zinc	0.53
Iron	17.13	Nickel	0.61	Iron	0.98	Nickel	0.12
Tin	4.66	Lead	0.33	Tin	0.62	Lead	0.14
Aluminum	1.04	Silver	0.16	Aluminum	5.52	Silver	NA

Notice: The content of silver is expressed as “g/t”. Because the content of silver is very low, the content of silver in the sample is tested by atomic absorption spectrometry.

**Table 6:** Test factors and levels of high-gradient magnetic separation tests.

Code	Factors	Units	Minimum	Maximum	Levels
A	Magnetic flux density	T	0.912	1.185	3
B	Pulse intensity	n/min	100	300	3
C	Particle size	mm	−0.125	−0.5	3

**Table 7:** Integrated evaluation of goodness of the recommended model in high-gradient magnetic separation tests.

Standard Deviation	0.64	R^2^	0.9936
Mean value	74.65	Correcting R^2^	0.9853
Deviation coefficient	0.85	Predicting R^2^	0.9882
Prediction error sum of squares	5.21	Predicting residual sum of squares	39.072

**Table 8:** The results of high-gradient magnetic separation under optimal conditions.

Optimal Conditions	Magnetic Field (T)	Pulse Intensity (n/min)	Particle Size (mm)	MA (Fe %)			NMA (Cu %)		
				Yield	Grade	Recovery	Yield	Grade	Recovery
	0.912	100	0.25	26.62	73.42	78.11	73.38	70.17	74.02

**Table 9:** The elements distribution of NMA and MA of high-gradient magnetic separation.

NMA				MA			
Element	Grade/(wt%)	Element	Grade/(wt%)	Element	Grade/(wt%)	Element	Grade/(wt%)
Copper	70.17	Zinc	3.69	Copper	8.54	Zinc	1.94
Iron	1.03	Nickel	0.15	Iron	73.42	Nickel	1.79
Tin	5.57	Lead	0.08	Tin	2.18	Lead	1.01
Aluminum	1.05	Silver	0.1	Aluminum	0.66	Silver	0.19

Notice: The content of silver is expressed as “g/t”. Because the content of silver is very low, the content of silver in the sample is tested by atomic absorption spectrometry.

## Data Availability

The data presented in this study are available on request from the corresponding author.

## References

[1] Amato A., Becci A., Beolchini F. (2020). Sustainable recovery of Cu, Fe and Zn from end-of-life printed circuit boards. Resour. Conserv. Recycl..

[2] Arshadi M., Yaghmaei S., Esmaeili A. (2020). Evaluating the optimal digestion method and value distribution of precious metals from different waste printed circuit boards. J. Mater. Cycles Waste Manag..

[3] Barnwal A., Dhawan N. (2020). Recovery of Metals from Discarded Integrated Circuits. Min. Metall. Explor..

[4] Golev A., Corder G.D., Rhamdhani M.A. (2019). Estimating flows and metal recovery values of waste printed circuit boards in Australian e-waste. Miner. Eng..

[5] Awasthi A.K., Li J. (2017). Management of electrical and electronic waste: A comparative evaluation of China and India. Renew. Sust. Energ. Rev..

[6] Szalatkiewicz J. (2016). Metals Recovery from Artificial Ore in Case of Printed Circuit Boards, Using Plasmatron Plasma Reactor. Materials.

[7] Hao J., Wang Y., Wu Y., Guo F. (2020). Metal recovery from waste printed circuit boards: A review for current status and perspectives. Resour. Conserv. Recycl..

[8] Tian S., Luo Y., Chen J., He H., Chen Y., Ling Z. (2019). A Comprehensive Study on The Accelerated Weathering Properties of Polypropylene-Wood Composites with Non-Metallic Materials of Waste-Printed Circuit Board Powders. Materials.

[9] Huang Y.-F., Lo S.-L. (2020). Energy recovery from waste printed circuit boards using microwave pyrolysis: Product characteristics, reaction kinetics, and benefits. Environ. Sci. Pollut. Res..

[10] Zhu L., Zhang M., He J., Liu C., Yao Y., Xu J., Xu X. (2021). Recovery of metal fractions from waste printed circuit boards via the vibrated gas-solid fluidized bed. Adv. Powder Technol..

[11] Yao Y., Zhou K., He J., Zhu L., Zhao Y., Bai Q. (2021). Efficient recovery of valuable metals in the disposal of waste printed circuit boards via reverse flotation. J. Cleaner Prod..

[12] Murugesan M.P., Kannan K., Selvaganapathy T. (2020). Bioleaching recovery of copper from printed circuit boards and optimization of various parameters using response surface methodology (RSM). Mater. Today-Proc..

[13] Baniasadi M., Graves J.E., Ray D.A., De Silva A.L., Renshaw D., Farnaud S. (2021). Closed-Loop Recycling of Copper from Waste Printed Circuit Boards Using Bioleaching and Electrowinning Processes. Waste Biomass Valoriz..

[14] Ippolito N.M., Medici F., Pietrelli L., Piga L. (2021). Effect of Acid Leaching Pre-Treatment on Gold Extraction from Printed Circuit Boards of Spent Mobile Phones. Materials.

[15] Yamane L.H., de Moraes V.T., Romano Espinosa D.C., Soares Tenorio J.A. (2011). Recycling of WEEE: Characterization of spent printed circuit boards from mobile phones and computers. Waste Manag..

[16] Qiu R.J., Lin M., Ruan J.J., Fu Y.G., Hu J.Q., Deng M.L., Tang Y.T., Qiu R.L. (2020). Recovering full metallic resources from waste printed circuit boards: A refined review. J. Clean. Prod..

[17] Ma F., Tao Y., Xian Y. (2021). Study of Enhanced Gravity Separation Based on Liberation Characteristics of a Heat-Treated Circuit Board. Min. Metall. Explor..

[18] Duan C., Wen X., Shi C., Zhao Y., Wen B., He Y. (2009). Recovery of metals from waste printed circuit boards by a mechanical method using a water medium. J. Hazard. Mater..

[19] Yang J., Wang H., Zhang G., Bai X., Zhao X., He Y. (2019). Recycling organics from non-metallic fraction of waste printed circuit boards by a novel conical surface triboelectric separator. Resour. Conserv. Recycl..

[20] Wu J., Li J., Xu Z. (2008). Electrostatic separation for recovering metals and nonmetals from waste printed circuit board: Problems and improvements. Environ. Sci. Technol..

[21] Huang Z., Zhu J., Qiu R.J., Ruan J.J., Qiu R.L. (2019). A cleaner and energy-saving technology of vacuum step-by-step reduction for recovering cobalt and nickel from spent lithium-ion batteries. J. Clean. Prod..

[22] Zhan L., Xu Z. (2009). Separating and recycling metals from mixed metallic particles of crushed electronic wastes by vacuum metallurgy. Environ. Sci. Technol..

[23] Silvas F.P.C., Jiménez Correa M.M., Caldas M.P.K., de Moraes V.T., Espinosa D.C.R., Tenório J.A.S. (2015). Printed circuit board recycling: Physical processing and copper extraction by selective leaching. Waste Manag..

[24] Oluokun O.O., Otunniyi I.O. (2020). Chemical conditioning for wet magnetic separation of printed circuit board dust using octyl phenol ethoxylate. Sep. Purif. Technol..

[25] Zhou G., Xu X., Wang S., He X., He W., Su X., Wong C.P. (2017). Surface grafting of epoxy polymer on CB to improve its dispersion to be the filler of resistive ink for PCB. Results Phys..

[26] Zhu P., Chen Y., Wang L., Qian G., Zhang W.J., Zhou M., Zhou J. (2013). Dissolution of brominated epoxy resins by dimethyl sulfoxide to separate waste printed circuit boards. Environ. Sci. Technol..

[27] Kumar A., Holuszko M.E., Janke T. (2020). Determination of loss on ignition test conditions for nonmetal fraction from processed waste printed circuit boards. Resour. Conserv. Recycl..

[28] Kang M.-S., Kim D.-S., Shin Y.-E. (2019). The Effect of Epoxy Polymer Addition in Sn-Ag-Cu and Sn-Bi Solder Joints. Materials.

[29] Rudajevova A., Dusek K. (2018). Influence of Manufacturing Mechanical and Thermal Histories on Dimensional Stabilities of FR4 Laminate and FR4/Cu-Plated Holes. Materials.

[30] Tao P., Chen Y., Cai W., Meng Z. (2021). Effect of Copper Sulfate and Sulfuric Acid on Blind Hole Filling of HDI Circuit Boards by Electroplating. Materials.

[31] Panda R., Jadhao P.R., Pant K.K., Naik S.N., Bhaskar T. (2020). Eco-friendly recovery of metals from waste mobile printed circuit boards using low temperature roasting. J. Hazard. Mater..

[32] Yi X., Qi Y., Li F., Shu J., Sun Z., Sun S., Chen M., Pu S. (2019). Effect of electrolyte reuse on metal recovery from waste CPU slots by slurry electrolysis. Waste Manag..

[33] Meng L., Guo L., Guo Z. (2019). Separation of metals from metal-rich particles of crushed waste printed circuit boards by low-pressure filtration. Waste Manag..

[34] Liu F.F., Wan B.B., Wang F.Z., Chen W.P. (2019). Effect of thermal shock process on the microstructure and peel resistance of single-sided copper clad laminates used in waste printed circuit boards. J. Air Waste Manag..

[35] Khammar S., Bahramifar N., Younesi H. (2020). Optimization using the response surface methodology for adsorption of polychlorinated biphenyls (PCBs) from transformer oil by magnetic CMCD-Fe3O4@SiO2 nanoparticles. Mater. Chem. Phys..

[36] Zhang Z., Yan G., Zhu G., Zhao P., Ma Z., Zhang B. (2020). Using microwave pretreatment to improve the high-gradient magnetic-separation desulfurization of pulverized coal before combustion. Fuel.

